# Chlorine inhalation-induced ARDS treated with noninvasive ventilation and awake prone positioning: a case report

**DOI:** 10.3389/fmed.2026.1926703

**Published:** 2026-09-09

**Authors:** Yu E. Xue, Jianyue Zhang, Zanfeng Cao, Zuopeng Zhang

**Affiliations:** 1 Department of Emergency Medicine, The First Affiliated Hospital of Guangzhou Medical University, Guangzhou, China; 2 Guangzhou Institute of Emergency Medicine, Guangzhou Medical University, Guangzhou, China

**Keywords:** acute respiratory distress syndrome, chlorine, high-flow nasal cannula, noninvasive mechanical ventilation, prone positioning

## Abstract

Chlorine-containing disinfectants are widely used as household cleaning agents and in swimming pools and industrial settings. Accidents involving these disinfectants are an important public health concern. Exposure to high chlorine concentrations can induce acute chlorine toxicity, with acute poisoning from chlorine gas primarily affecting the respiratory system and causing irritation of the eyes, nose, and throat, and occasionally the skin. Significant exposure may result in acute respiratory distress syndrome (ARDS), which carries a risk of mortality. This report describes the case of a 76-year-old man who developed chlorine inhalation-induced ARDS after exposure to toxic gas generated from a trichloroisocyanuric acid disinfectant in a swimming pool. On admission, his PaO_2_/FiO_2_ ratio was 184 mmHg, meeting the Berlin definition of ARDS. He presented with progressive dyspnea and bilateral pulmonary infiltrates, and recovered completely after treatment using noninvasive mechanical ventilation, awake prone positioning, glucocorticoids, and comprehensive supportive treatment. As there is no specific antidote for chlorine poisoning, management focuses on symptomatic treatment, prevention of complications, and close observation until the patient is clinically stable. This case shows that early recognition and comprehensive supportive treatment, including noninvasive ventilation and awake prone positioning, can improve oxygenation, prevent intubation, and achieve favorable clinical outcomes.

## Introduction

Chlorine gas is a potent respiratory irritant that can cause severe lung injury following accidental exposure. Trichloroisocyanuric acid, a common disinfectant for swimming pools, releases chlorine gas upon contact with water. While the majority of exposures result in mild, self-limiting symptoms, high-concentration or prolonged exposure can lead to ARDS. We present a case of moderate chlorine-induced ARDS successfully managed with noninvasive ventilation and awake prone positioning, highlighting practical strategies to avoid intubation.

### Case presentation

A 76-year-old man was admitted to our hospital on May 31, 2025, with a seven-hour history of recurrent dyspnea. Approximately seven hours before admission, the patient had been swimming in a public swimming pool when he inhaled toxic gas produced by a trichloroisocyanuric acid–based disinfectant. His location at the time of inhalation was approximately 10 meters from the disinfectant source, and the estimated duration of exposure was 10 min. Shortly after exposure, the patient developed recurrent shortness of breath, accompanied by an irritating dry cough, dizziness, and headache. He was immediately transferred to an open, well-ventilated area, resulting in partial alleviation of the symptoms. However, his condition subsequently deteriorated, with progressive dyspnea accompanied by facial flushing and profuse sweating. He was therefore transferred to our hospital, where he was admitted to the Emergency Intensive Care Unit (EICU) for further management. The patient's past medical history included coronary artery disease, hypertension, and type 2 diabetes mellitus. There was no relevant personal or family history of respiratory disease.

**Figure 1:**
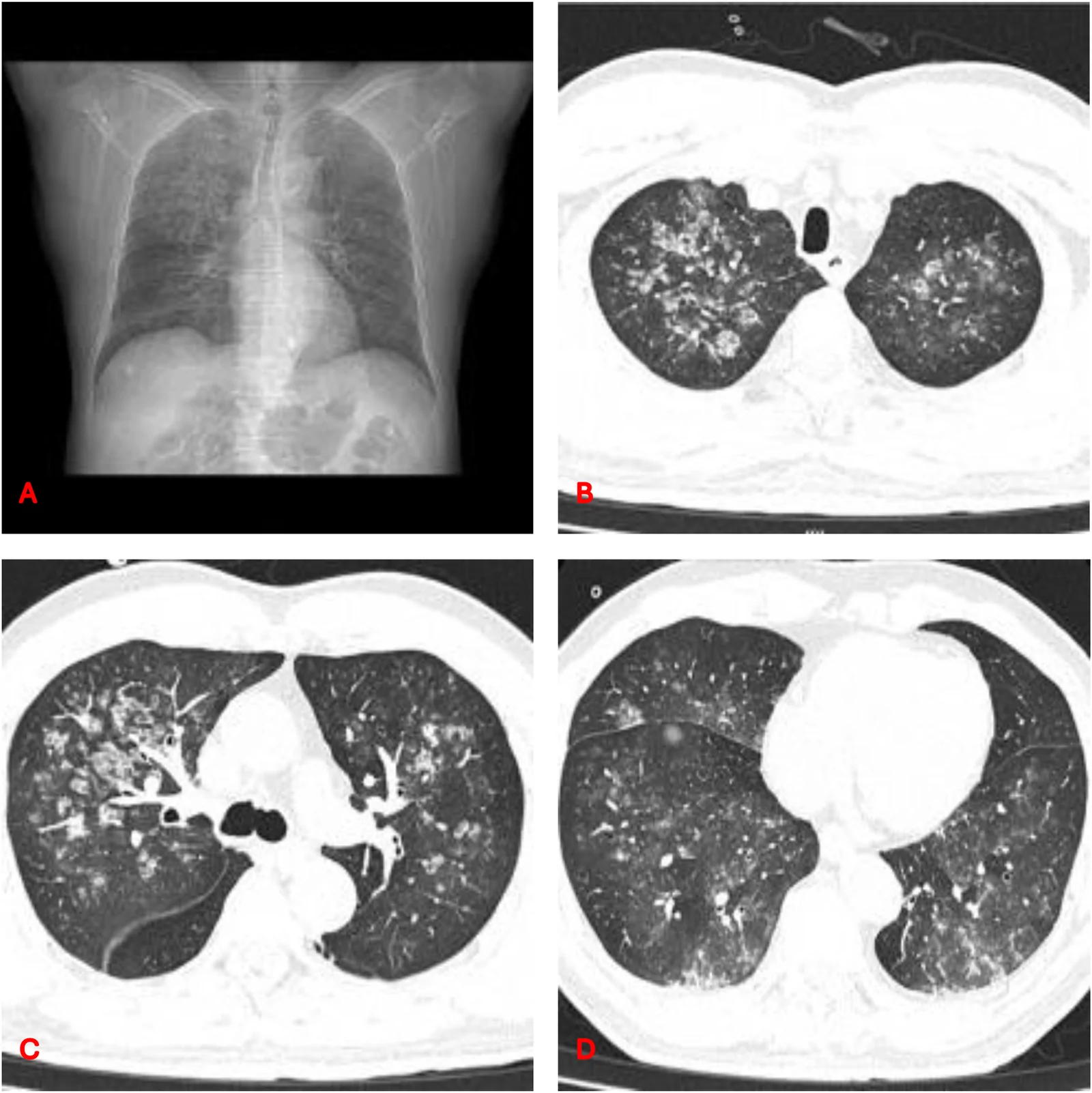
Initial chest x-ray and CT findings before treatment in chlorine inhalation–induced ARDS. **(A)** Chest x-ray on admission. **(B–D)** Corresponding chest CT images obtained on admission.

On admission, the patient's vital signs were stable, with a temperature of 36.5 °C, pulse rate of 88 bpm, respiratory rate of 35 breaths/min, blood pressure of 128/75 mmHg, and SpO₂ of 100%. Physical examination showed coarse bilateral breath sounds with scattered wheezes, without moist crackles. Cardiac examination was unremarkable. Analysis of arterial blood gas indicated a pH of 7.368, PaCO₂ of 39.6 mmHg, PaO₂ of 73.6 mmHg, and an oxygenation index (PaO₂/FiO₂) of 184 (Table 1). Chest computed tomography (CT) showed multiple bilateral pulmonary infiltrates, consistent with inflammatory changes and possible pulmonary edema (Figure 1).

**Table 1:** Laboratory parameters and oxygenation indices during hospitalization.

	D1	D2	D3	D4	D5	D6	D7	D8	D9	D10	D11
White Blood Cell	11.44	12.47	14.22	10.56	9.28	6.84	6.52	7.51	6.50	11.40	7.68
Neutrophils	10.43	11.03	13.06	9.51	7.9	5.79	4.71	5.4	4.48	9.11	5.61
Lymphocyte	0.59	1.08	0.65	0.59	0.85	0.62	1.28	1.59	1.49	1.55	1.51
Red Blood Cell	7.35	7.26	6.07	5.88	5.99	5.51	5.77	6.02	5.98	5.71	5.77
Hemoglobin	150	147	124	118	121	111	116	121	121	115	114
Platelet	224	92	189	198	184	173	168	176	156	197	190
Procalcitonin	0.059			0.095			0.052				0.057
pH	7.368	7.35	7.382	7.414	7.39	7.39	7.431	7.427	7.425	7.423	7.355
pCO_2_	39.6	43	39.4	35	35.8	41.7	40.6	40.1	39.4	40	48.7
pO_2_	73.6	181.4	102.5	72.6	156.2	183.9	148	148.3	92.2	115.7	105.4
HCO3-	22.2	23.1	22.9	22	21.2	24.7	26.5	25.9	25.4	25.7	26.5
ABE	−2.3	−2.1	−1.5	−1.5	−2.7	0.2	2.5	2	1.5	1.7	0.9
SBE	−2.3	−1.7	−1.5	−1.9	−3.0	0.3	2.6	2	1.4	1.7	1.5
SBC	22.4	22.7	23.2	23.1	22.1	24.6	26.7	26.2	25.7	25.9	25.2
PaO₂/FiO₂ ratio	184	302	204	145	312	368	370	344	184	319	234
Respiratory support	S/T	S/T HFNC	S/T HFNC	CPAP HFNC	CPAP HFNC	CPAP HFNC	CPAP HFNC	HFNC	HFNC	6L O_2_	6L O_2_
FiO_2_(%)	40	60	50	50	50	50	40	43	50	36	45

Imaging revealed diffuse bilateral ground-glass opacities with multifocal patchy consolidations involving both lungs. The abnormalities were predominantly distributed along the bronchovascular bundles and in the dependent lung regions, consistent with acute toxic inhalation lung injury complicated by ARDS. The extensive bilateral involvement correlated with the patient's severe hypoxemia at presentation.

Based on the exposure history, acute onset, Chest imaging demonstrate bilateral pulmonary opacities that cannot be fully explained by pleural effusion, lung collapse, or pulmonary nodules, a PaO₂/FiO₂ ratio of 184 mmHg with a minimum PEEP of 5 cmH₂O, an NT-proBNP level of 222 pg/mL, and transthoracic echocardiography showing no significant structural or functional cardiac abnormalities, cardiogenic pulmonary edema was excluded. The patient therefore met the diagnostic criteria for ARDS according to the Berlin definition and was subsequently diagnosed with moderate ARDS secondary to chlorine gas poisoning.

**Figure 2:**
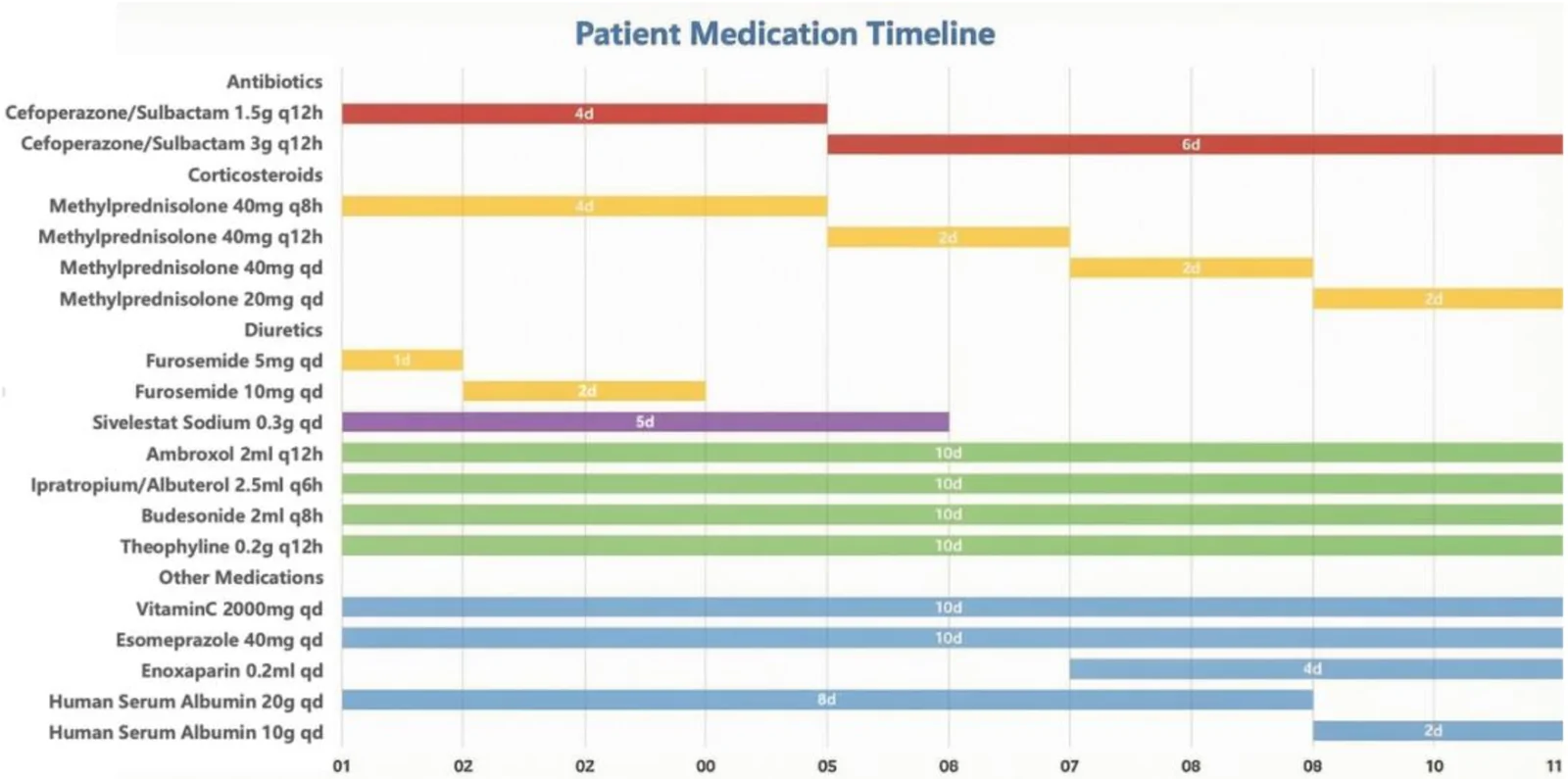
Patient medication timeline.

**Figure 3:**
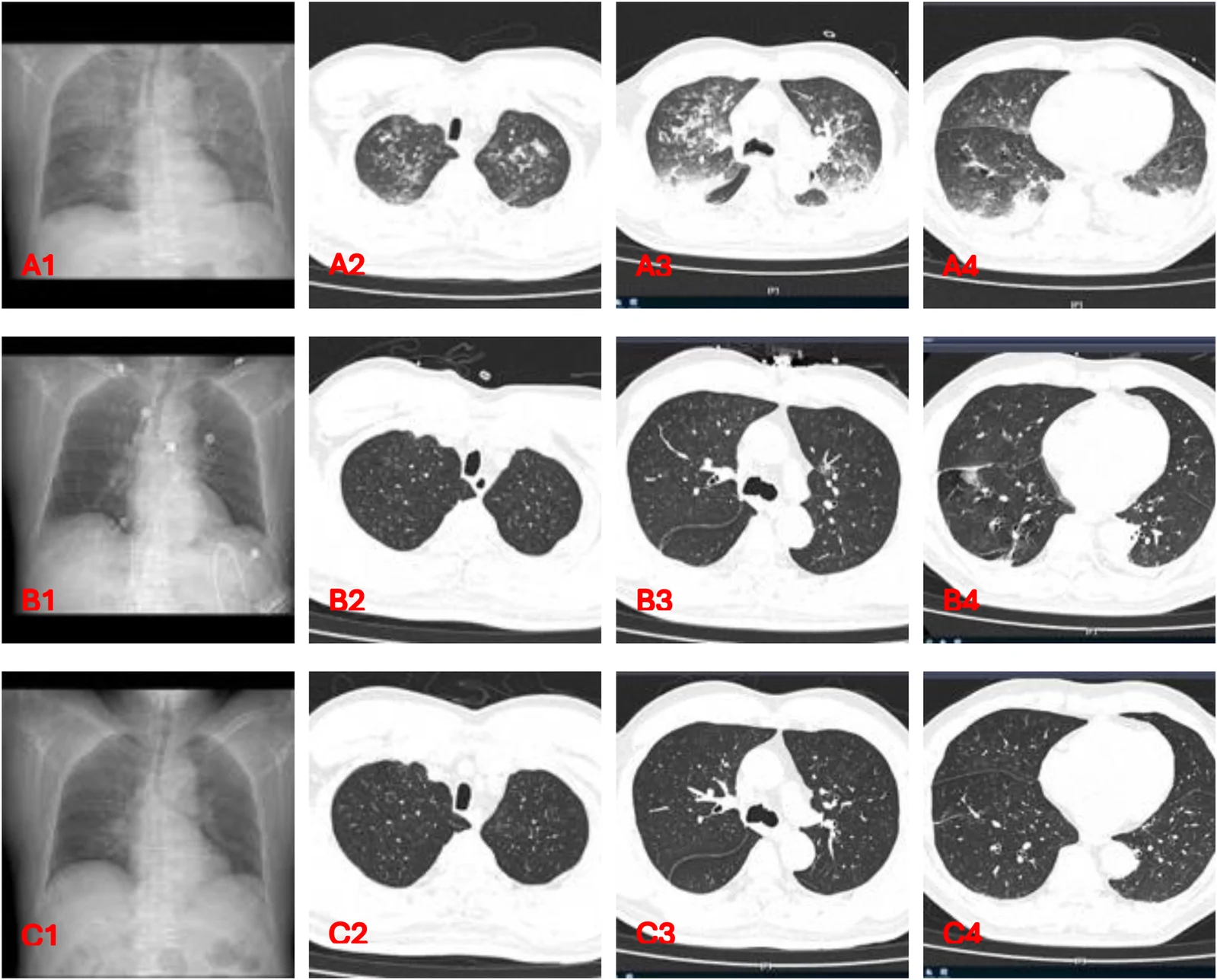
Chest CT findings after treatment for chlorine inhalation–induced ARDS. **(A1–C1)** Chest x-ray on Day 4, Day 10, and Day 26. **(A1–A4)** Day 4 imaging demonstrated diffuse bilateral ground-glass opacities and patchy consolidations, predominantly in the dependent and lower lung regions. **(B1–B4)** Day 10 imaging showed marked regression of pulmonary opacities with improved lung aeration. **(C1–C4)** Day 26 imaging revealed near-complete resolution of the previous abnormalities, indicating substantial recovery of chlorine inhalation–induced lung injury.

Following admission, the patient received noninvasive mechanical ventilation (NIV) using S/T mode with an inspiratory positive airway pressure (IPAP) of 12 cmH₂O, an expiratory positive airway pressure (EPAP) of 5 cmH₂O, and a respiratory rate of 16 breaths/min, patient symptomate improve, continuous positive airway pressure (CPAP) with a PEEP of 5–8 cmH₂O, alternating with high-flow nasal cannula (HFNC) therapy at 40–50 L/min during NIV breaks. Awake prone positioning was initiated on the second day of admission, performed three times daily for 30 min per session, over three consecutive days. Cefoperazone-sulbactam was administered empirically for antimicrobial coverage. Airway clearance techniques, including chest physiotherapy and breathing exercises, were applied. Systemic corticosteroid therapy with methylprednisolone was administered using a tapering regimen: 40 mg every eight hours between days 1 and 4, 40 mg twice daily from days 5 to 7, 40 mg once daily on days 8 and 9, and 20 mg once daily on days 10 and 11. In addition, sivelestat sodium was used for its anti-inflammatory and lung-protective effects. Oxygen-driven nebulization therapy (ambroxol hydrochloride, ipratropium bromide, doxofylline, budesonide) was administered (Figure 2). Strict fluid management was maintained with a negative fluid balance, together with gastrointestinal protection and glycemic control. The respiratory symptoms resolved completely, and the patient was discharged in stable condition with no residual clinical symptoms. Follow-up chest CT showed complete resolution of pulmonary infiltrates (Figure 3). Informed consent was obtained from the patient for the publication of this case report and accompanying images.

## Discussion

**Figure 4:**
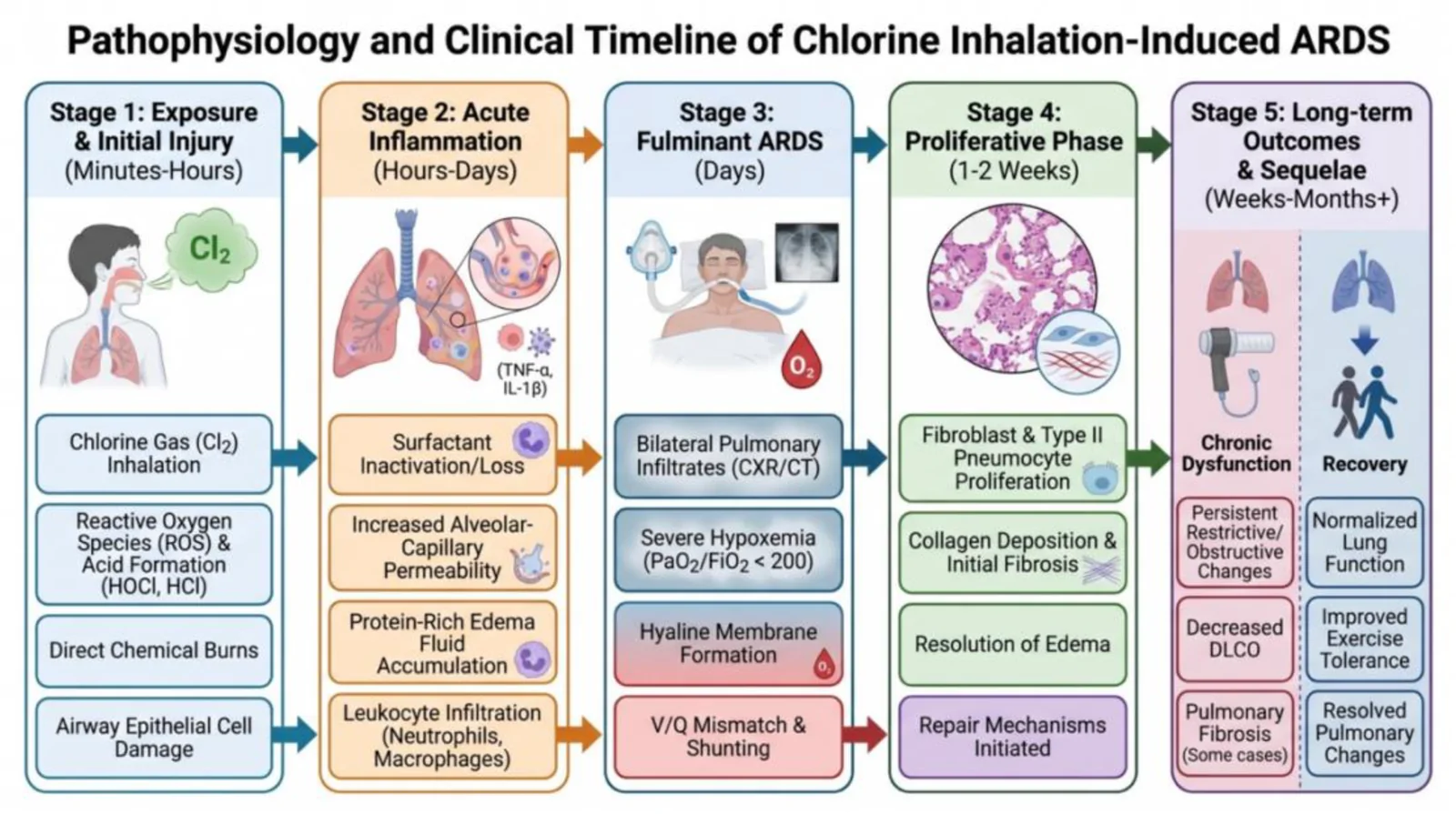
Pathophysiology and clinical timeline of chlorine inhalation-induced ARDS.

Trichloroisocyanuric acid is widely used to disinfect swimming pools and public spaces due to its potent antimicrobial properties (1). However, it releases chlorine gas upon contact with water, resulting in a significant risk of inhalation if proper safety protocols are not followed (2). The inhaled chlorine reacts rapidly with water in the airway lining fluid to form hydrochloric acid and hypochlorous acid, causing oxidative damage to airway and alveolar epithelial cells. This leads to disruption of epithelial tight junctions and compromises the integrity of the alveolar-capillary barrier. The initial epithelial damage triggers a cascade of inflammatory responses, characterized by neutrophil recruitment and activation. These activated neutrophils release reactive oxygen species, proteases, and pro-inflammatory cytokines, further amplifying tissue injury and endothelial dysfunction (3). Severe exposure can increase microvascular permeability, leading to leakage of protein-rich fluid into the alveolar spaces, which induces non-cardiogenic pulmonary edema (Figure 4). The combination of these processes culminates in diffuse alveolar damage and the clinical manifestation of ARDS (4).

The patient inhaled a large amount of chlorine gas generated from trichloroisocyanuric acid over a short period, resulting in acute injury to the lungs and bronchi and subsequent respiratory distress. Non-contrast chest CT performed on the day of admission indicated multiple bilateral pulmonary infiltrates. According to the Berlin definition of ARDS, an acute onset following inhalation of a known inhalational insult, the presence of bilateral opacities on chest imaging, and a minimum PaO₂/FiO₂ ratio of 180 mmHg with a minimum positive end-expiratory pressure of 5 cmH₂O support the diagnosis of ARDS after exclusion of cardiogenic pulmonary edema.

Protective ventilation and supportive respiratory strategies remain the cornerstone of ARDS management (5). In the present case,NIV was initiated using S/T and CPAP, with HFNC therapy applied during intermittent NIV breaks. PEEP minimizes alveolar collapse and reopening, reducing ventilator-induced lung injury (6). HFNC complements NIV by delivering high-flow, heated, and humidified oxygen, enhancing comfort and tolerance. HFNC also reduces anatomical dead space, facilitates clearance of secretions, and provides a modest PEEP effect, further supporting alveolar recruitment and gas exchange. A study by Frat et al. found that patients with nonhypercapnic acute hypoxemic respiratory failure, high-flow oxygen, standard oxygen, and noninvasive ventilation had similar intubation rates, with a modest difference in 90-day mortality favoring high-flow oxygen (7). The combined use of NIV and HFNC represents a lung-protective strategy that optimizes oxygenation while avoiding risks associated with invasive mechanical ventilation, awake patients may have difficulty tolerating or cooperating with NIV, and HFNC provides an alternative respiratory support option. Intubation would have been considered if the patient developed persistent or worsening hypoxemia despite optimized non-invasive respiratory support, severe respiratory distress or increased work of breathing, hemodynamic instability, deterioration in consciousness or inability to protect the airway, or other signs of clinical deterioration.

Glucocorticoids exert anti-inflammatory effects by binding to glucocorticoid receptors and modulating the expression of pro-inflammatory mediators. In addition, they may promote alveolar fluid clearance by upregulating epithelial sodium channel (ENaC) expression and enhancing active sodium reabsorption, thereby potentially reducing pulmonary edema. Their use is conditionally recommended in patients with ARDS (8, 9). In the case, methylprednisolone led to improved respiratory status and reduced pulmonary edema on bedside lung ultrasound. Inhalational injury may disrupt the airway epithelial barrier and impair mucociliary clearance, thereby increasing the risk of secondary bacterial pneumonia. Given the potential for secondary infection in patients with inhalational injury, empirical cephalosporin therapy was initiated early. Furthermore, neutrophil elastase, a key mediator of vascular endothelial injury and increased vascular permeability, plays a central role in ARDS pathogenesis (10). Sivelestat sodium, a selective neutrophil elastase inhibitor, may be associated with improved oxygenation in early ARDS (11). Awake prone positioning can improve oxygenation by increasing the ventilation/perfusion ratio and promoting dorsal lung re-expansion while preserving spontaneous breathing. Early application under close monitoring can alleviate dyspnea and delay or avoid endotracheal intubation.

In addition to ARDS, the patient developed transient thrombocytopenia, with the platelet count decreasing from 224 × 10⁹/L to 92 × 10⁹/L and subsequently recovering to 189 × 10⁹/L. This transient change may have been related to the acute inflammatory response and coagulation activation associated with chlorine-induced lung injury. Experimental studies have reported thrombocytopenia following chlorine exposure, along with activation of coagulation pathways and pulmonary microthrombus formation. However, the exact mechanism remains uncertain, and other causes of transient platelet consumption or dilution cannot be excluded (12).

Avoidance of chlorine gas exposure remains the most effective preventive strategy. Treatment of ARDS is primarily supportive and multidisciplinary, emphasizing early oxygen therapy,lung-protective ventilation, and comprehensive management, including prone positioning, anti-infective therapy, and corticosteroids. Prompt intervention is crucial to improve outcomes. Chest CT is a valuable for anatomical assessment but is not an ideal indicator of immediate treatment response.

## Data Availability

The datasets presented in this article are not readily available because of ethical and privacy restrictions. Requests to access the datasets should be directed to the corresponding author.
